# Impact of completing a psychosocial rehabilitation programme on inpatient service utilisation in South Africa

**DOI:** 10.4102/sajpsychiatry.v28i0.1764

**Published:** 2022-10-21

**Authors:** Yanga Vava, Liezl Koen, Dana Niehaus, Henmar F. Botha, Ulla Botha

**Affiliations:** 1 Department of Psychiatry, Faculty of Medicine and Health Sciences, Stellenbosch University, Cape Town, South Africa

**Keywords:** deinstitutionalisation, psychosocial rehabilitation, transitional care, mental illness, days-in-hospital

## Abstract

**Background:**

Deinstitutionalisation refers to the process of transferring most of the psychiatric care provision from inpatient state-run institutions to community-based care. However, it has proven difficult to implement and failed to reach its desired targets. New Beginnings (NB) is a transitional care facility that facilitates the transition from in- to outpatient care. To date, no data exist as to whether the intervention provided at NB is effective in reducing psychiatric readmissions.

**Aim:**

To determine if completing a psychosocial rehabilitation (PSR) programme reduces acute inpatient service utilisation and if this is influenced by sociodemographic or clinic factors.

**Setting:**

New Beginnings transitional care facility in South Africa.

**Methods:**

A record review of all NB admissions between January 2011 and December 2015. Demographic and clinical data were collected, including readmissions and days-in-hospital (DIH), 36 months pre- and postindex admission. Patients were divided into a completer group (CG) and a noncompleter group (NCG) for the eight-week PSR programme, and comparative statistical analysis was performed.

**Results:**

Completion of the 8-week voluntary inpatient PSR programme led to a significant decrease (*p* = 0.017) (CG vs. NCG) in DIH during the 36-month period postindex admission. In addition, both groups showed significantly decreased (*p* < 0.001) DIH postindex in comparison to pre-index admission.

**Conclusions:**

This study’s findings support that transitional care facilities offering an inpatient PSR programme may reduce inpatient service utilisation for all attendees but especially for those who complete the program. This highlights the need for such facilities that offer interventions tailored for patients with mental illness.

**Contribution:**

This is the first local study highlighting the potentially important role transitional care facilities could play in reducing readmissions.

## Introduction

Deinstitutionalisation refers to the global changes in mental health policy that prompted the move of mentally ill patients out of state-run psychiatric institutions to community-based services.^1^ This process began in the middle to late 20th century, partially prompted by an increase in asylum populations found to be associated with overcrowding and in some instances abuse of patients.^2^ Deinstitutionalisation was driven by the assumption that community-based care would be more humane, therapeutic and cost-effective compared to hospital management.^1^ In South Africa, the concept of deinstitutionalisation was introduced after the 1994 democratic elections, when primary health care became the central focus for the restructuring of health services.^3^

However, deinstitutionalisation has proven difficult to implement and has failed to reach desired targets, both in South Africa and abroad.^4^ One problem has been that, while the number of psychiatric beds was rapidly decreased, the necessary community-based resources were not made available to meet the demand. This led to severe bed pressures, resulting in an increase in the number of premature discharges from psychiatric hospitals. In this context, premature discharges refer to mental health care users who are discharged before they are fully stabilised. This has led to reduced recovery and poorer outcomes, as patients are vulnerable to early relapse and subsequent readmission, or the so-called ‘revolving door’ pattern of care, because of community-based services unable to cope with the growing numbers.^1,2,5^

Revolving door patterns are further exacerbated by other challenges such as stigma associated with mental illness, comorbid substance use, poor treatment adherence and inadequate preparation of caregivers to provide the necessary postdischarge support.^1,6,7^ Overall, these multiple challenges contributed to increased readmission rates in acute settings.

Proper discharge planning is the first step towards improved outcomes, but in pressured inpatient units this process is often rushed or neglected. Hengartner et al.^8^ emphasised the need for carefully planned and coordinated transition from in- to outpatient care. There have been several publications reporting on transitional care interventions, both internationally and locally.^9,10,11,12^ In their systematic review, Steffen et al.^10^ concluded that ‘discharge interventions are effective in reducing rehospitalisation and improving adherence to aftercare among people with mental disorders’. The interventions reported on in the review included telephonic reminders or letters, assignment of care coordinators, compiling of pharmacy discharge plans and an outpatient clinician or aftercare group contact facilitation prior to discharge.

Locally, a randomised controlled trial conducted by Botha et al.^11^ assessed the impact of a modified assertive intervention and demonstrated a significant reduction in readmissions and days-in-hospital (DIH). The authors later assessed the influence of a telephone-based intervention on readmission rate in patients with severe mental illness and found no significant impact on inpatient service utilisation.^13^ Brooke-Sumner et al.^14^ looked at psychosocial groups facilitated by social workers in the North West province of South Africa and reported benefits of the programme. Some of the challenges reported included issues with supply of antipsychotic medication and lack of appropriate venue, highlighting the importance of transitional care facilities.

Transitional care facilities are community-based, subacute, recovery-focused services which can be used as step-up (admission from the community) or step-down (from inpatient units) facilities. These facilities offer patients more time to recover from their illness, psychosocial rehabilitation (PSR) and education for patients and carers regarding postdischarge care, which improves the chance of a smooth transition from in- to outpatient care.^12^ Thomas et al.^12^ assessed symptoms, functioning and quality of life after treatment in a residential subacute mental health service in Australia. Step-down patients reported improvements in symptoms and functioning, and the authors concluded that these transitional facilities may offer a cost-effective alternative to hospital admissions. To date, there is a paucity of information related to the effectiveness of transitional care facilities in resource-limited countries, including South Africa.

New Beginnings (NB) is a transitional care facility in South Africa. This 40-bed step-up–step-down (SUSD) unit was launched in 2008 by the Department of Health in the Western Cape province with a mandate to reduce pressure on psychiatric inpatient units in the Northern Substructure catchment area and to improve quality of care. New Beginnings offers an eight-week structured voluntary PSR-based programme which aims to facilitate transitional care, allowing more time for recovery and optimisation of treatment.^15^

The NB PSR programme focuses on activities that help patients prepare for independent living with additional emphasis on substance use intervention and recovery. Core features of the programme also include medication adherence and insight-orientated therapy to foster independence in clients and encourage active involvement in decision-making. In collaboration with nongovernmental organisations, some patients also participate in internship programmes for work placement. On completion of the programme, patients are discharged to their families or caregivers and mostly follow-up at their local community health centres. Monthly support groups are available postdischarge from NB to support clients and carers with medication adherence, substance use and family concerns, as well as matters relating to their general well-being.

A recent publication describes the demographic and clinical profile of patients who accessed the NB facility between 2011 and 2015.^15^ However, no data exist as to whether the intervention provided at this transitional care facility is effective in reducing psychiatric readmissions. We conducted a retrospective record review of patients admitted to NB to determine if completion of the eight-week PSR programme influenced readmission rates and overall inpatient service utilisation and if so, whether this was influenced by sociodemographic or clinical factors. The data obtained in this study may be useful for informing future mental health service planning initiatives.

## Methods

### Study design

We conducted a retrospective record review of patients admitted to NB between 01 January 2011 and 31 December 2015. This period was selected to allow for a postinclusion period of 36 months.

### Study setting

New Beginnings is a SUSD facility situated in the Western Cape, South Africa. The facility was launched in 2008 and offers a structured eight-week, voluntary PSR-based programme which aims to facilitate the transition from in- to outpatient care, giving more time for recovery and optimisation of treatment.

The programme offers exercise groups; education on mental illness, medication, comorbid substance abuse and general well-being; life skills groups; daily chores for independent living; craft projects to stimulate creativity; volunteer groups; and recreational drumming. Caregivers are engaged as part of the programme to ensure their readiness to take on their role. A full description of the programme as well as the inclusion criteria was detailed in the publication by Botha et al.^15^

### Study sample

All male and female patients (*n* = 730) who were admitted to NB from 01 January 2011 to 31 December 2015 were eligible for inclusion. Patients receiving follow-up treatment from the local assertive community treatment team (*n* = 69) and those who had multiple admissions (two or more) (*n* = 132) to NB were excluded. An additional 20 patients were removed from the final dataset because of incomplete or missing data. The final sample size for analysis was 509 patients (327 noncompleters and 182 completers).

### Data collection

Data were collected from the NB patient folders as well as Clinicom Application Manager (Western Cape hospital database keeping record of patient demographics, outpatient appointments and hospital admissions). All data were collated in a Microsoft Excel spreadsheet. Demographic and clinical data, including data on the duration of stay at NB, completion of the NB programme and subsequent readmissions and DIH, were collected for the 36-month periods pre- and postindex admission to the programme.

### Data analysis

Continuous variables were summarised as mean (or median where appropriate) and standard deviation (s.d.), while nominal variables were summarised as counts and percentages. Patients were divided into a completer group (CG) and a noncompleter group (NCG) of the eight-week PSR programme. Pearson’s chi-square test (Fisher’s exact test where appropriate) and *t*-test for equality of means were used to test for differences in selected demographic and clinical variables between the two groups (CG and NCG). Independent-samples Mann–Whitney U test was used to assess differences in DIH pre- and postindex admission between groups. Related-samples Wilcoxon signed-rank tests were performed to compare mean DIH 36 months pre- and postdischarge from NB after the index admission for both the groups. SPSS version 26 software was used for all analyses, and statistical significance was set at *p* < 0.05.

### Ethical considerations

Ethical approval was obtained from the Health Research Ethics Committee of Stellenbosch University (reference number S19/01/025), and a waiver of informed consent was granted because of the retrospective nature of the study. All data were anonymised to ensure confidentiality of patients’ personal information, with each patient assigned a unique identifier.

## Results

### Demographic characteristics

Five hundred and nine patients (mean age 27.92 years, s.d.: 8.17, range 18–59 years) were included in the study. Most patients were male (*n* = 414, 81.3%), single (*n* = 465, 91.4%), educated to a grade 7–9 level (*n* = 173, 34.0%), unemployed (*n* = 463, 90.9%) and not receiving a disability grant (DG) (*n* = 367, 72.1%). Table 1 summarises the comparison of the demographic characteristics between the CG and the NCG. Only age differed significantly between the groups, with the CG being slightly older (28.87 years vs. 27.38 years, *p* = 0.048, 95% confidence interval [CI]: between − 2.972 and − 0.016).

**TABLE 1:** Demographic characteristics’ comparison (noncompleter group vs. completer group) of the patients included in our study (*n* = 509).

Variable	Overall		Completion of new beginnings programme, *n* (%)				Difference between NCG and CG
			Noncompleters, < 8 weeks (*n* = 327)		Completers, ≥ 8 weeks (*n* = 182)		*p*
	*n*	%	*n*	%	*n*	%	
**Gender**							0.156
Male	414	81.3	272	83.2	142	78.0	–
Female	95	18.7	55	16.8	40	22.0	–
**Marital status**							0.172
Single	465	91.4	304	93.0	161	88.5	–
Divorced	22	4.3	9	2.8	13	7.1	–
Married	17	3.3	11	3.4	6	3.3	–
Widowed	4	0.8	2	0.6	2	1.1	–
Unknown	1	0.2	1	0.3	0	0.0	–
**HLOE**							0.919
Grade 1–6	42	8.3	26	8.0	16	8.8	–
Grade 7–11	282	55.4	170	52.0	112	61.6	–
≥ Grade 12	111	21.8	65	19.9	46	25.3	–
Unknown	74	14.5	66	20.2	8	4.4	–
**Emplo yment**							0.566
Unemployed	463	90.9	296	90.5	167	91.8	–
Employed	46	9.1	31	9.5	15	8.2	–
**Disability grant**							0.303
No	367	72.1	241	73.7	126	69.2	–
Yes	142	27.9	85	26.3	56	30.8	–
**Addre ss**							0.913
Cape Town metro area	451	88.6	292	89.3	159	87.3	–
Rural (outside metro area)	58	11.4	35	10.7	23	12.7	–
**Primary carer**							0.876
Family	476	93.5	306	93.6	170	93.4	–
Spouse	9	1.8	6	1.8	3	1.6	–
Self	8	1.6	5	1.5	3	1.6	–
Other	16	3.1	10	3.1	6	3.3	–
**Accom modation**							0.762
Family	483	94.9	310	94.8	173	95.0	–
Own	8	1.6	4	1.2	4	2.2	–
Rent	7	1.4	5	1.5	2	1.1	–
Shelter	6	1.2	5	1.5	1	0.5	–
Group	5	1.0	3	0.9	2	1.1	–

HLOE, highest level of education; NCG, noncompleter group; CG, completer group.

### Clinical characteristics

Table 2 summarises the comparison of the clinical characteristics between the CG and the NCG (*n* = 509). The community mental health nurse was identified as the primary health care provider for most of the patients (*n* = 497, 97.6%). The primary diagnosis for most patients was schizophrenia (*n* = 264, 51.9%). The only difference that could be demonstrated between the two groups was that patients from the NCG were significantly more likely (*p* = 0.04) to use illicit substances.

**TABLE 2:** Clinical characteristics’ comparison (noncompleters vs. completers) of the patients included in our study (*n* = 509).

Variable	Overall		Completion of new beginnings programme				Difference between NCG and CG
			Noncompleters, < 8 weeks (*n* = 327)		Completers, ≥ 8 weeks (*n* = 182)		*p*
	*n*	%	*n*	%	*n*	%	
**Primary health care provider**							0.477
Community mental health nurse	497	97.6	319	97.6	178	97.8	–
Psychiatric hospital	6	1.2	3	0.9	3	1.6	–
Private psychiatrist	6	1.2	5	1.5	1	0.5	–
**Primary psychiatric diagnosis**							0.197
Schizophrenia	264	51.9	162	49.5	102	56.0	–
Substance-induced psychotic disorder	122	24.0	87	26.6	35	19.2	–
Bipolar mood disorder	62	12.2	44	13.5	18	9.9	–
Schizoaffective disorder	40	7.9	23	7.0	17	9.3	–
Other	12	24.0	7	2.1	5	2.7	–
Major depressive disorder	9	1.8	4	1.2	5	2.7	–
**Illicit substances**							0.04*
None used	126	24.8	72	22.0	54	29.7	–
Used illicit substances	378	74.4	255	78.0	127	69.8	–
**Alcohol use disorder**							0.651
No	467	91.7	298	91.1	169	92.9	–
Yes	42	8.3	29	8.9	13	7.1	–
**Medical condition or treatment**							0.063
No	441	86.6	291	89.0	150	82.4	–
Single medical condition	61	12.0	31	9.5	30	16.5	–
Multiple medical conditions	7	1.4	5	1.5	2	1.1	–

* , Significant at a level of *p* < 0.05.

NCG, noncompleter group; CG, completer group.

### Clinical outcomes

#### Number of admissions

There was no significant difference (*p* = 0.83, 95% CI: -0.202 to 0.251) in the number of admissions pre-index between CG (*n* = 182; mean 1.89) and NCG (*n* = 327; mean 1.91). There was also no significant difference (*p* = 0.07, 95% CI: -0.022 to 0.508) in the number of admissions postindex between CG (*n* = 182; mean 0.93 admissions) and NCG (*n* = 327; mean 1.17 admissions).

#### Days-in-hospital

**FIGURE 1:**
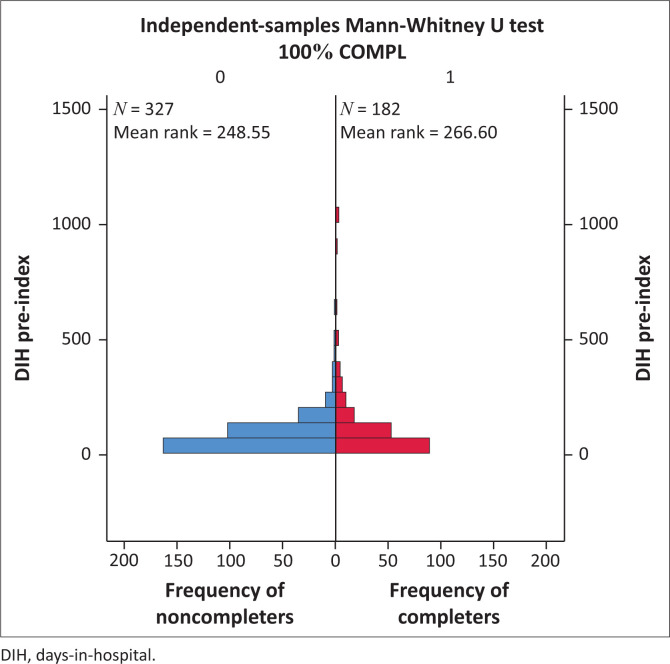
Comparison of days-in-hospital pre-index for completer group versus noncompleter group.

**FIGURE 2:**
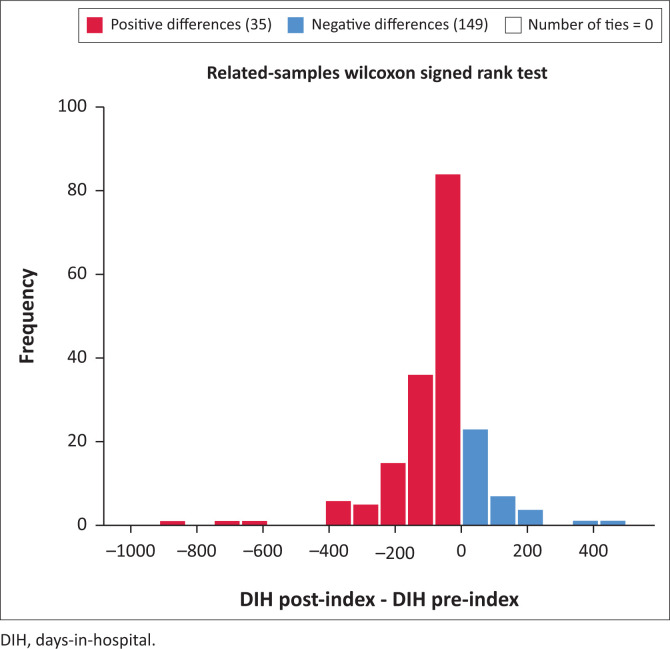
Comparison of pre- and postindex days-in-hospital for completer group.

**FIGURE 3:**
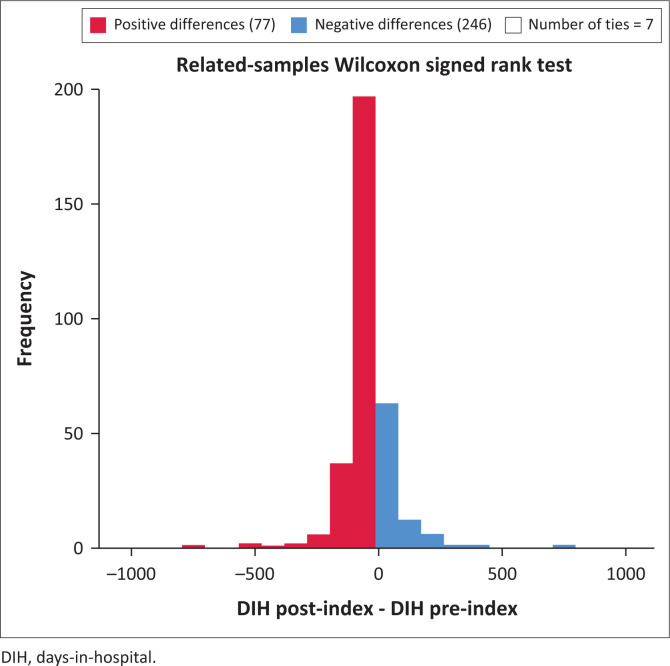
Comparison of pre- and postindex days-in-hospital for noncompleter group.

**FIGURE 4:**
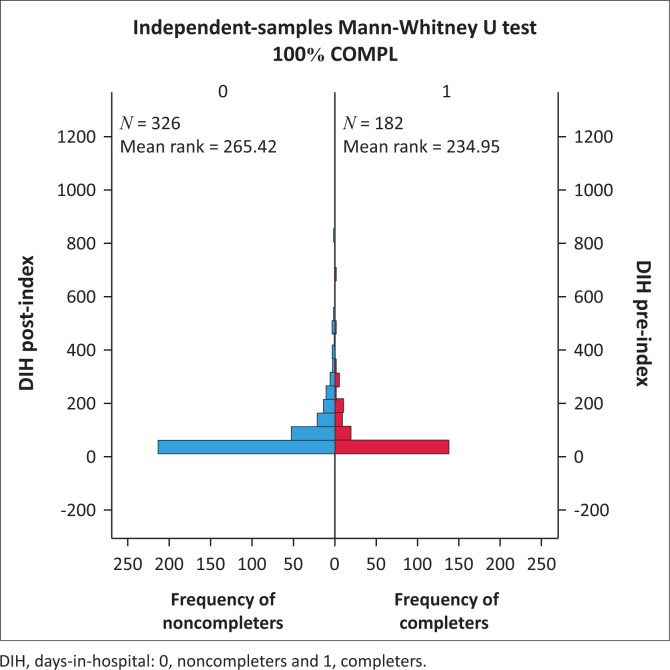
Comparison of days-in-hospital postindex for completer group versus noncompleter group.

Days-in-hospital pre-index did not differ significantly for the comparison between CG and NCG (116.9 pre vs. 93.5 post) (*p* = 0.184) (Figure 1). Both groups did, however, show a significant decrease in DIH when pre- and postindex admissions were compared. For CG see Figure 2 (116.9 days decreased to 41.34 days, *p* < 0.001) and for NCG see Figure 3 (93.5 days decreased to 55.05 days, *p* < 0.001). Furthermore, there was a significant difference in the number of DIH postindex admission between the two groups, with the CG spending significantly fewer DIH (41.34 vs. 55.05) (*p* = 0.017) (Figure 4).

## Discussion

This study’s findings show that completion of an 8-week voluntary inpatient PSR programme at the NB SUSD transitional care facility led to a significant decrease in DIH during the 36-month period postindex admission for the CG in comparison to the NCG. Furthermore, both the CG and the NCG showed significantly decreased DIH postindex admission in comparison to pre-index admission.

New Beginnings provides recovery and rehabilitation services through a short-term programme. The programme runs for eight weeks to accommodate all the interventions offered at the facility, which includes, among others, psychoeducation and skills training. The facility initially (year 1) utilised more flexible rehabilitation periods, but because of several factors, they decided on an eight-week programme. Factors contributing to this decision included a high demand for rehabilitation beds, and periods longer than 12 weeks were experienced as counterproductive. Prior to this study, no data existed on the effectiveness of the programme, and our findings suggest that NB does reduce inpatient service utilisation. Owing to the high demand for the programme as well as the lack of resources, it seems that the 8-week duration is adequate to rehabilitate patients and allows NB to receive new patient groups every two months for rehabilitation and in so doing relieve pressures on inpatient service utilisation.

Age was the only sociodemographic variable that differed significantly between the groups, with the CG being slightly older, which could tie in with the fact that the NCGs were shown to be more likely to use illicit substances. A previous study in South Africa showed that cannabis and other drugs (including cocaine and extramedical drugs) were more commonly used in younger age groups (18–29 years old).^16^ Furthermore, more than 60% of those in the 18–29 years age group had used drugs (stimulants, cocaine, etc.) by the age of 17 years, while none had used these drugs by the age of 17 years in individuals who were 50 years or older. Peltzer and Phaswana-Mafuya^17^ also showed that, among others, being of younger age was associated with any drug use in the past 3 months, and according to the 2020 (Phase 49) South African Community Epidemiology Network on Drug Use (SACENDU) report,^18^ most patients receiving treatment for substance use in South Africa were younger than 30 years of age, with patients in this age group accounting for 70% in the Northern region (Mpumalanga and Limpopo), 67% in the Eastern Cape, 63% in Gauteng, 55% in KwaZulu-Natal and 41% in the Western Cape.

The NB programme has very strict regulations about substance use, and patients are often tested on their return from weekend leave. Patients who are found to have used substances during their stay are asked to leave the programme. Although reasons for noncompletion were not explored in this study, it is likely that this policy contributed to this, with patients either being asked to leave or signing themselves out to access substances. The main concern would still lie in the overall high rates of illicit substance use reported in this population, which is in keeping with that documented for a similar local population.^19^

It is well documented that substance use behaviour can be associated with more psychotic symptoms, treatment nonadherence and increased cost of care and higher relapse rates.^20,21^ Looking at the sample from a gender perspective, the rates are in keeping with use reported to be more common among male patients.^22^ One explanation that has been offered for this ‘gender gap’ is that the culture and policies that influence the access to and acceptability of substance use favour men.^23^ The low rate of alcohol use disorder in this study may be attributed to under-reporting. Patients and families may not consider alcohol a substance of abuse, given that it is legal and used widely.^24^ Overall, these findings once again emphasise the need for substance use interventions that are tailored for patients with severe mental illness. As there is very limited access to any such dual diagnosis inpatient programmes, it should be critically considered how transitional facilities such as NB could help address this gap.

As aforementioned, most of the patients admitted to NB were male. This could be explained in part that the facility has more male than female beds, with 32 and 8, respectively, in keeping with identified pressures as related to the inpatient units. Other local studies have also supported the male predominance in acute inpatient units in South Africa.^19,25^

Most patients resided in the community with family members. Caregiver burden in psychiatric illness in comparison to chronic medical illness has been shown to be higher, which could be associated with the caregiver’s own risk of mental illnesses such as depression, anxiety and burnout.^26^ This underlines the importance of comprehensive discharge planning with focused family engagement. New Beginnings offers this as part of the PSR programme to improve readiness to take on the caregiver role and to facilitate access to other support services.^15^

Community mental health services were identified as the primary longitudinal care providers. These services are heavily burdened, and patients can often not be seen regularly due to large caseloads.^13^ A programme such as that offered by NB highlights the critical nature of bridging the transition between in- and outpatient care to provide time for more effective communication and engagement with primary care, which may alleviate some of the pressure on community health services.

Our study also found that a large percentage of patients were unemployed and not receiving a DG. The low percentage of DGs may be explained by applications still being processed or that some patients did not meet the criteria for receiving such. It is also possible that some information in this regard may be incomplete, as these data are not formally captured on Clinicom in a structured manner. It is well known that patients with serious mental illness who are not fully stabilised struggle to find or maintain employment.^27^ Other obstacles faced by patients in their pursuit for employment include stigma, patient attitudes and self-esteem.^28^ High unemployment rates most often correlate with poor socio-economic status, which has been associated with medication nonadherence and its implications for maintaining illness stability.^29^ Some patients at NB are afforded the opportunity to participate in internship programmes for work placement. This is encouraging as vocational rehabilitation approaches can lead to employment for some patients.^30^

Interestingly, both the CG and NCG demonstrated to have significantly reduced inpatient usage (DIH) postindex NB admission, suggesting that access to NB brings benefits. However, the CG group still performed significantly better than the NCG, which probably highlights the true gains if a patient can fully access a voluntary and appropriately structured intervention, where patients are required to follow a structured routine with enough time (in the case of this study, eight weeks) for proper discharge planning and engagement in psychosocial and recovery interventions. In addition, this emphasises the need to make these voluntary interventions more acceptable to patients to support them in completing the programme. This may take the form of establishing a proper therapeutic relationship with the patient, properly assessing the needs of the patient’s prior referral to the transitional facility, assessing their motivation for change and psycho-educating them properly on their illness as well as medication adherence. It is also important to acknowledge that patients with a history of illicit substance use may find it more difficult to complete the programme, and ways should be explored to engage them more effectively. With this in mind, NB now offers a specialised dual diagnosis programme. The programme differs from traditional treatment approaches in that it focuses on preventing anxiety and improving coping, rather than breaking through the denial. Also, patients do not have to want to achieve sobriety in order to attend; they simply need to be willing to talk about it. The goal of the programme is for clients to achieve total abstinence; however, any reduction in harmful use is seen as a move in the right direction.

## Limitations

To facilitate data analysis, patients who had multiple admissions were excluded. This was a retrospective study that depended mainly on information documented in patient folders and recorded on the Clinicom database. Results from this study may not be generalisable to all transitional care facilities.

## Conclusion

This study’s findings demonstrate that transitional care facilities offering an inpatient PSR programme may significantly reduce inpatient service utilisation for all attendees of such, with those who completed the full 8-week programme faring significantly better. This highlights the need for more such facilities that offer interventions tailored for patients with serious mental illness.
